# Hyperthermic ablation by UltraSound Guided High Intensity Focused Ultrasound (USgHIFU) plus Systemic Chemotherapy (SC) for locally advanced pancreatic cancer: the secret of longer survival

**DOI:** 10.1186/2050-5736-2-S1-A6

**Published:** 2014-12-10

**Authors:** Joan Vidal-Jove, Eloi Perich, Marta Garcia-Bernal, Manuel Alvarez del Castillo

**Affiliations:** 1 Hospital University Mutua Terrassa, Barcelona, Spain

## 

We describe results in unresectable pancreatic tumors treated with USgHIFU hyperthermia ablation plus adjuvant chemotherapy.

## Materials and methods

Forty three cases of non resectable pancreatic tumors were treated from March 2010 to March 2013, and all of them underwent systemic chemotherapy. Clinical responses (thermical ablation achieved) were measured by image techniques. They were 29 Stage III cases and 14 Stage IV cases. Complications were also analyzed.

## Results

Clinical responses (ablation obtained) were 82% in all cases, sustained at 8 weeks of the procedure. We obtained 10 complete responses (25%) at the end of the combined treatment, 9 from stage III patients and 2 from stage IV. Major complications included severe pancreatitis with GI bleeding (1), skin burning grade III that required plastic surgery (2). No deaths were registered. Median Survival was 13 month (6 mo - 2.7 year).

## Conclusion

HIFU plus SC is a potentially effective and safe modality for the treatment of unresectable pancreatic cancer.

